# Supplementary figures and images for: Scorpionfish BPI is highly active against multiple drug-resistant Pseudomonas aeruginosa isolates from people with cystic fibrosis
Source: eLife. 2023 Jul 18;12:e86369. doi: 10.7554/eLife.86369 (PMC10353861; doi:10.7554/eLife.86369)

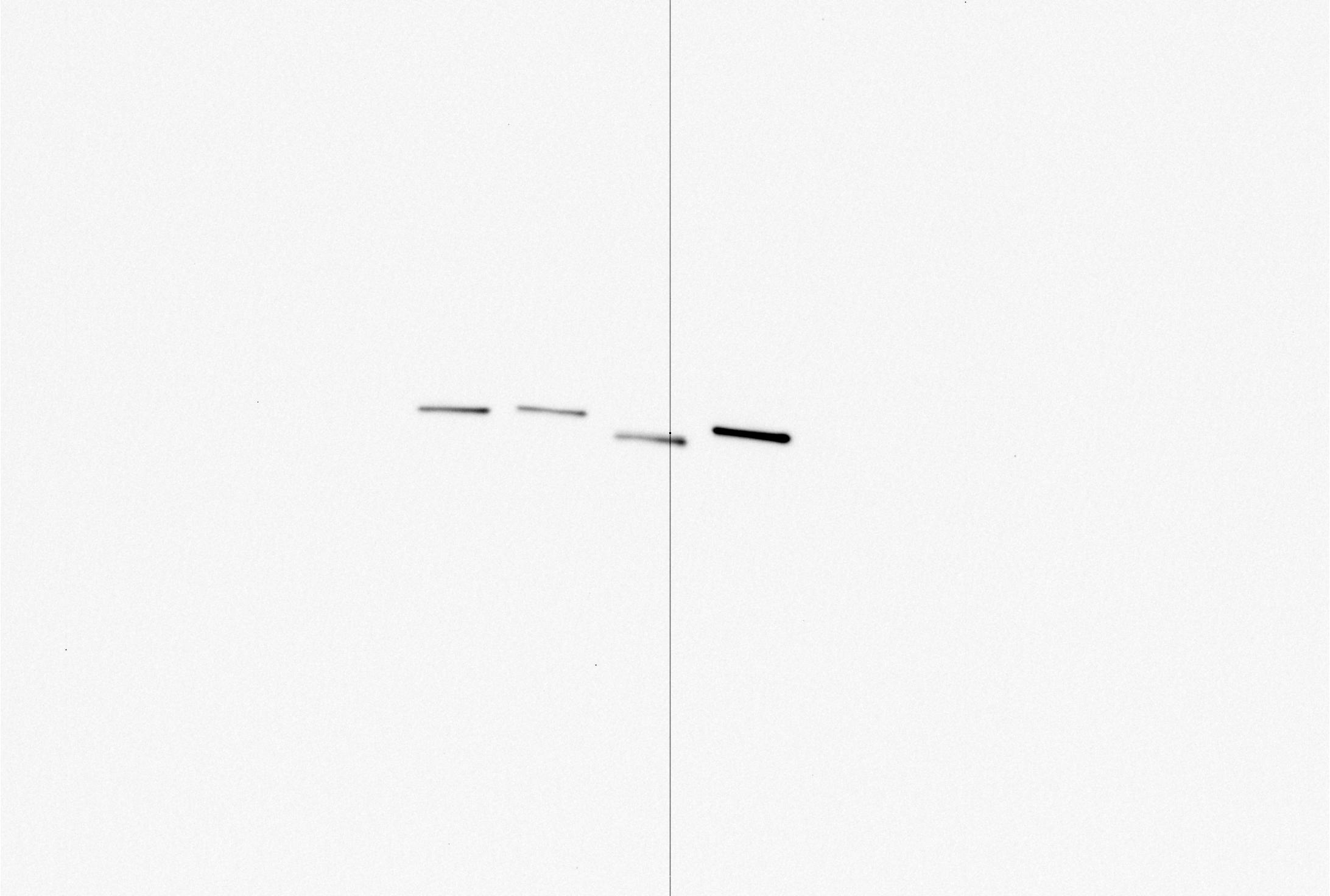

Supplement: Figure 2—source data 1. — Uncropped and labeled blots for Figure 2D. [file elife-86369-fig2-data1.zip › Figure 2D anti-FLAG raw blot.jpg]

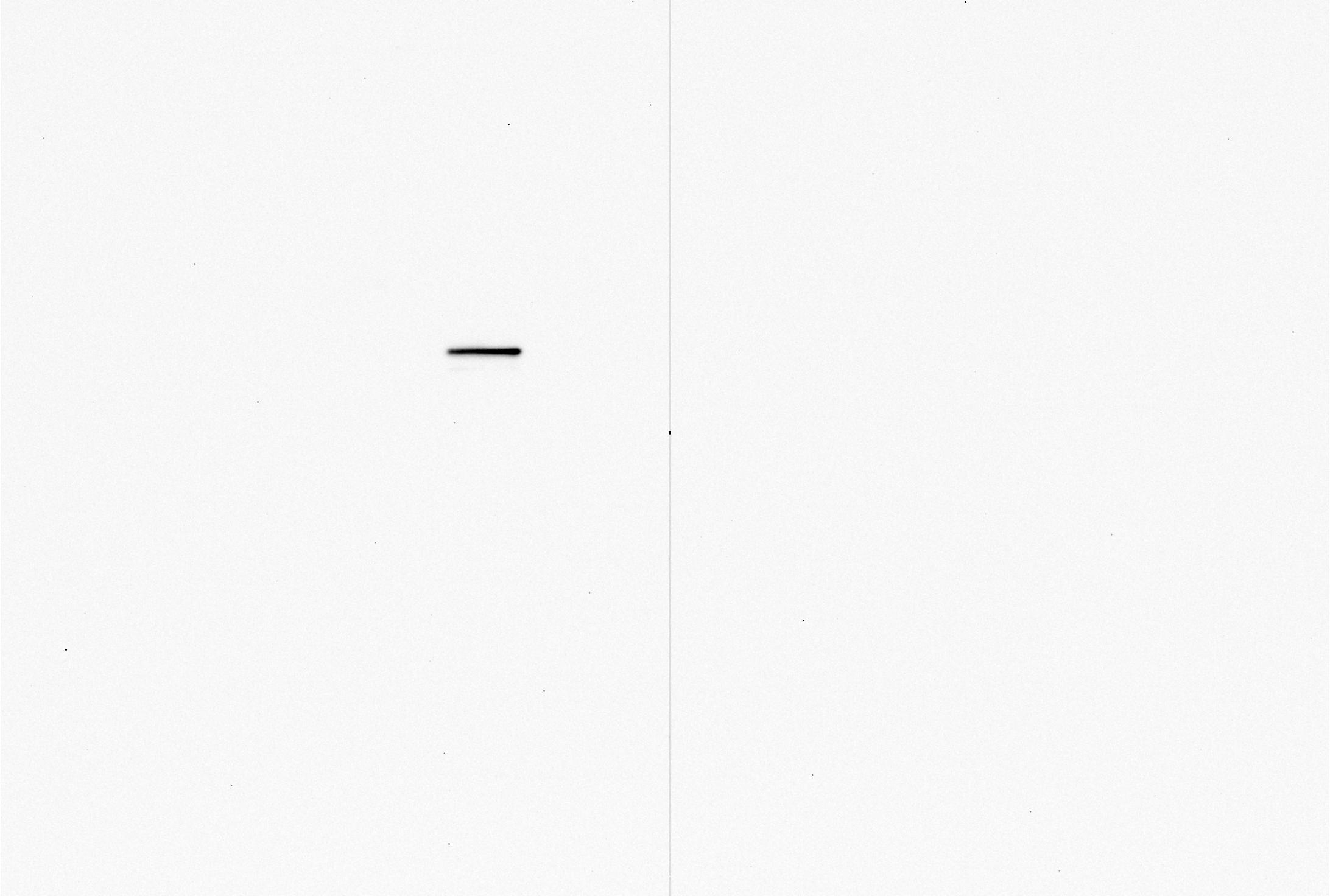

Supplement: Figure 2—source data 1. — Uncropped and labeled blots for Figure 2D. [file elife-86369-fig2-data1.zip › Figure 2D anti-huBPI raw blot.jpg]

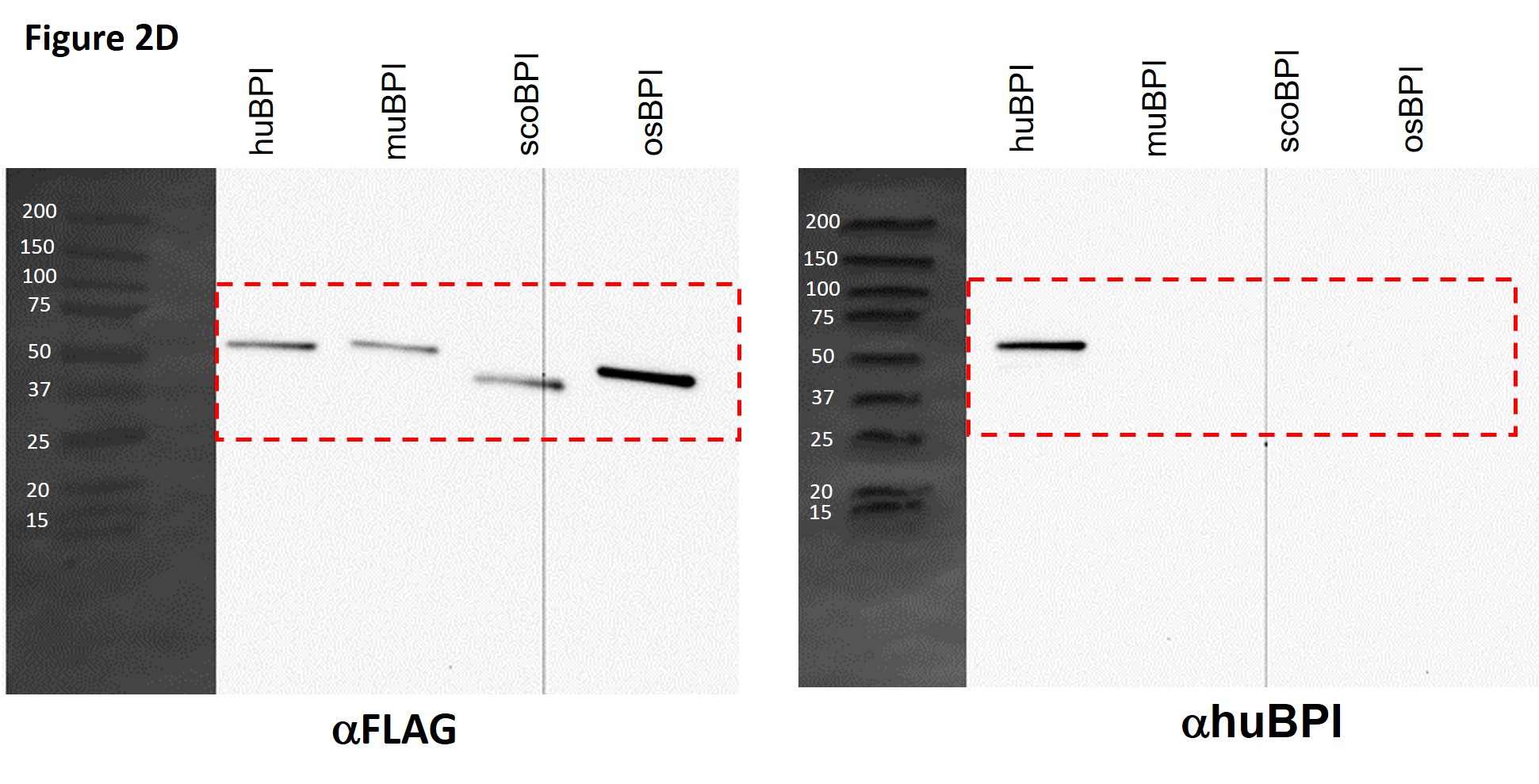

Supplement: Figure 2—source data 1. — Uncropped and labeled blots for Figure 2D. [file elife-86369-fig2-data1.zip › Figure 2D Blot uncropped gels.jpg]

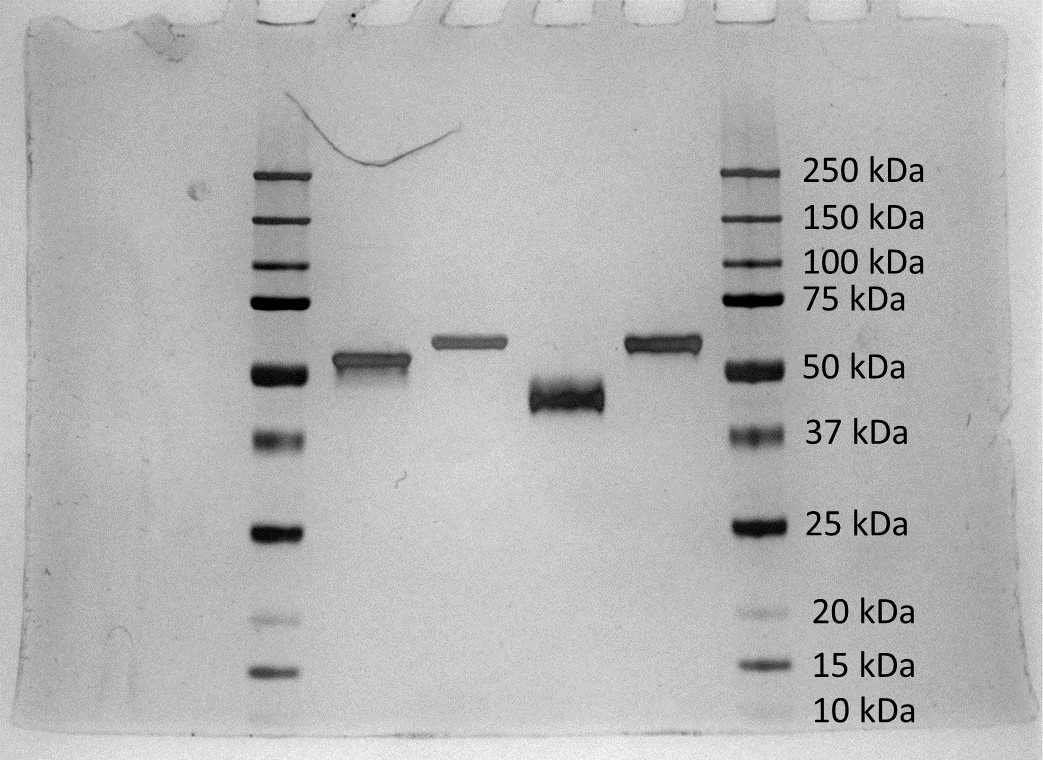

Supplement: Figure 2—figure supplement 1—source data 1. — Raw data for Figure 2—figure supplement 1D. [file elife-86369-fig2-figsupp1-data1.zip › Figure 2-figure supplement 1C labeled gel.jpg]

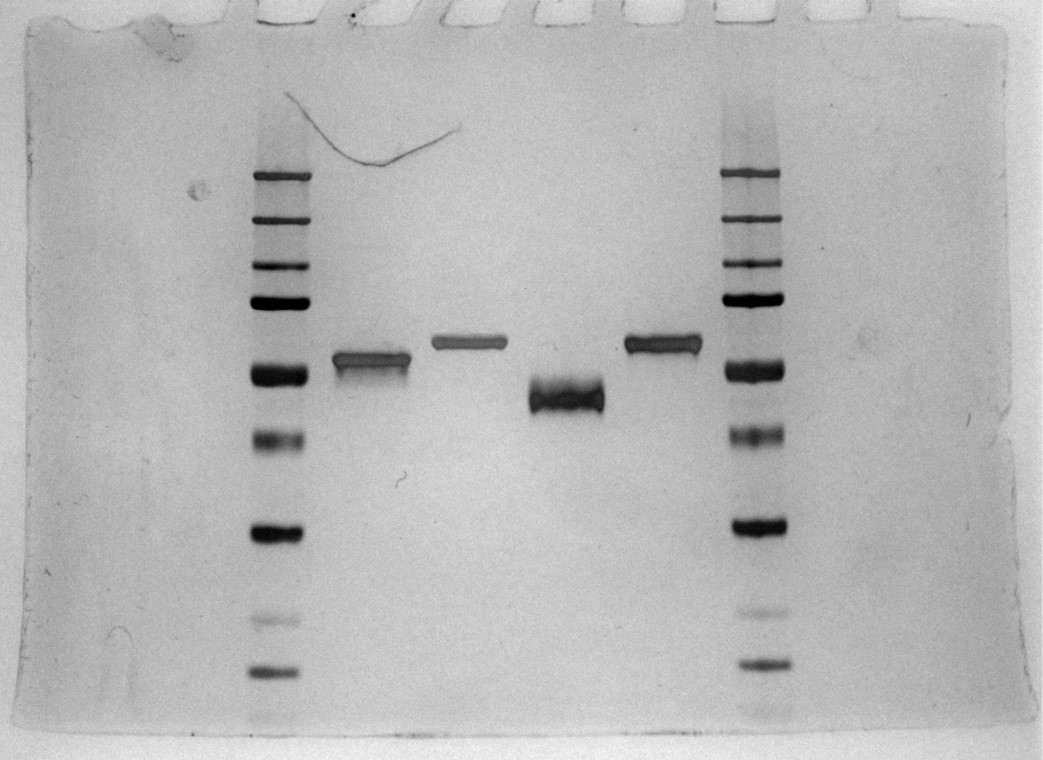

Supplement: Figure 2—figure supplement 1—source data 1. — Raw data for Figure 2—figure supplement 1D. [file elife-86369-fig2-figsupp1-data1.zip › Figure 2-figure supplement 1C uncropped gel.jpg]
